# Is It Better to Mobilize Hematopoietic Stem Cells With Pegfilgrastim in Healthy Donors During Allogeneic Hematopoietic Stem Cell Transplantation?

**DOI:** 10.3389/fonc.2020.01598

**Published:** 2020-09-03

**Authors:** Jiali Li, Sanbin Wang, Yicheng Zhang, Shifeng Lou, Yao Liu, Peiyan Kong, Cheng Zhang, Lei Gao, Xiangui Peng, Ping Wang, Xiaojuan Deng, Li Gao, Xi Zhang

**Affiliations:** ^1^Medical Center of Hematology, Xinqiao Hospital, Army Medical University, Chongqing, China; ^2^State Key Laboratory of Trauma, Burns and Combined Injury, Army Medical University, Chongqing, China; ^3^Department of Hematology, 920th Hospital of Joint Logistics Support Force, Yunnan, China; ^4^Department of Hematology, Tongji Hospital, Tongji Medical College, Huazhong University of Science and Technology, Wuhan, China; ^5^Department of Hematology, The Second Affiliated Hospital of Chongqing Medical University, Chongqing, China

**Keywords:** pegfilgrastim, peripheral blood, hematopoietic stem cells mobilization, allogeneic hematopoietic stem cell transplantation, single apheresis

## Abstract

The mobilization of hematopoietic stem cells (HSCs) using granulocyte colony-stimulating factor is a classic method. Recently, a single injection of pegfilgrastim was used to mobilize CD34+ cells in some small-sample studies. To confirm the efficacy and safety of pegfilgrastim in the mobilization of CD34+ cells from healthy donors, we conducted a retrospective multicenter study. A total of 146 healthy donors who all received subcutaneous pegfilgrastim (12 mg) on day 1 were enrolled in our study. Donor HSC apheresis was conducted on day 5. The primary endpoint was the percentage of donors from whom ≥4 × 10^6^ CD34+ cells/kg were collected in a single apheresis session. The median number of CD34+ cells in donors was significantly higher on day 5 than that on day 4 (82.26 μL vs. 51.65 μL, *P* ¡ 0.001). In 111 of the 146 donors, an optimal number of CD34+ cells (≥4 × 10^6^ kg) were collected in a single apheresis procedure. Bone pain and headache were the main adverse events, but the side effects did not require treatment. The number of white blood cells in most donors dropped to normal levels within 1 week after apheresis. Nearly 97% of patients achieved neutrophil and platelet engraftment. Pegfilgrastim for mobilization could be used to obtain an optimal number of CD34+ cells in a single session. Pegfilgrastim-induced mobilization not only was effective and safe but also avoided the pain of multiple injections and apheresis procedures in donors. However, prospective randomized controlled trials should be conducted in the future.

## Introduction

Allogeneic hematopoietic stem cell transplantation (allo-HSCT) is an important curative therapy for patients with malignant hematological neoplasms and non-malignant hematological disorders (1). Mobilization with granulocyte colony-stimulating factor (G-CSF) has been established as the standard regimen for HSCT for many years (2). However, the requirement for injections one to two times every day added to donor discomfort. More importantly, the optimal number of CD34+ cells, which was recommended to be 4–5 × 10^6^ CD34+ cells/kg by the American Society for Blood and Marrow Transplantation, could not be collected with one apheresis procedure in 37% of donors (3, 4). Therefore, some studies have tried to mobilize CD34+ cells in other new ways.

Recently, pegfilgrastim was introduced; pegfilgrastim has a longer elimination half-life and lower serum clearance than conventional G-CSF (5). Initially, pegfilgrastim was used as prophylaxis for chemotherapy-induced neutropenia (6). Recently, several preliminary studies in allogeneic donors showed the feasibility of mobilizing and harvesting CD34+ cells using pegfilgrastim (7–9). Donors were treated with single doses of 6 or 12 mg pegfilgrastim, and the efficiency of 12 mg pegfilgrastim for steady-state mobilization was investigated (8). However, the sample size of these studies was relatively small, and they did not focus on collecting the optimal number of CD34+ cells or the best time for harvesting.

Therefore, we retrospectively analyzed 146 healthy donors at four HSCT centers in China to evaluate the efficacy and safety of using a single 12-mg injection of pegfilgrastim for mobilization in allo-HSCT.

## Materials and Methods

### Study Criteria

This was a retrospective, single-arm study that was conducted at four HSCT centers in China. A total of 146 healthy donors who received a subcutaneous single dose of 12 mg pegfilgrastim (Qilu Pharmaceutical Co., Ltd, Shandong, China) for mobilization from October 2016 to September 2018 were enrolled in this study. A predonation examination was performed for all donors. These examination protocols included medical history, chest radiography, electrocardiography (ECG), complete blood counts, blood chemistry analysis, and infectious and immune marker screening.

The donors were required to fulfill these criteria: aged 18–60 years; weight from 45 to 100 kg; normal cardiac, liver, and kidney function; negative for human immunodeficiency virus, hepatitis B virus, and hepatitis C virus; and normal ECG, chest radiography, and abdominal ultrasonography examination findings. The exclusion criteria were as follows: unrelated allo-HSC donor; uncontrolled infection before mobilization; severe nervous system disorder affecting informed consent and/or adverse reactions; and hypertension, diabetes, and a history of ophthalmic-related diseases (such as retinal detachment).

### Endpoints and Definitions

The primary endpoint was the percentage of donors from whom ≥4 × 10^6^ CD34+ cells/kg were collected in a single apheresis procedure. Secondary endpoints included the side effects of mobilization, the proportion of donors who were able to mobilize ≥2 × 10^6^ CD34+ cells/kg with a single apheresis procedure, the number of peripheral blood (PB) CD34+ cells on day 4 and day 5, and the rates of graft-versus-host-disease (GVHD) and relapse. Neutrophil and platelet engraftment as well as outcomes were measured. The National Institutes of Health (NIH) Consensus (10, 11) criteria were used to grade acute GVHD (aGVHD) and chronic GVHD (cGVHD). Disease relapse was diagnosed on the basis of morphology or evidence of leukemic cells in either the bone marrow (BM) or other extramedullary organs. Progression-free survival (PFS) was defined as the shortest interval between HSCT and relapse or non-relapse mortality (NRM) or the last follow-up. Overall survival (OS) was defined as being alive at any time point.

### Donor Mobilization and Side Effects Assessments

**FIGURE 1:**
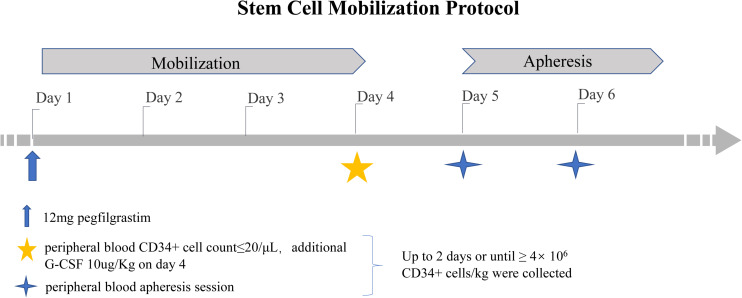
Biopsy examples.

Donors subcutaneously received a single dose of pegfilgrastim at 12 mg on day 1. If the number of PB CD34+ cells was ≤20 μL on day 4, an additional dose of 10 μg/kg G-CSF (Huabei Pharmaceutical Co., Ltd., Shijiazhuang, China) was administered. If the collection target was reached (≥4 × 10^6^ kg), apheresis was performed only on day 5. Otherwise, additional CD34+ cells were harvested on day 6 (Figure 1). CD34+ cell collection was performed using institution-standard apheresis procedures (3 blood volumes ±10%). The number of CD34+ cells was determined by flow cytometry. Laboratories were required to use either a BD Procount Progenitor Cell Enumeration Kit or a Beckman Coulter Stem Kit. The verification of laboratory proficiency was necessary, and the use of the CD-Chex CD34 product (Streck) to test the proficiency of the analysis was recommended (12).

All donors were requested to answer questionnaires about the side effects of the pegfilgrastim injection until 2 weeks after apheresis. The severity of the maximum pain was classified by the WHO toxicity criteria from 0 to 4 (0, no pain; 1, mild pain; 2, moderate pain; 3, severe pain; 4, very severe pain). Safety was evaluated based on changes from baseline in the medical history, biochemical index measurements, and physical examination findings.

### Transplantation Procedure

All transplant recipients received different conditioning regimens according to their diseases and transplant type. The conditioning regimen in aplastic anemia consisted of Fludara (Flu), Cyclophosphamide (CY), and Anti thymocyte globulin (ATG, Sanofi, SangStat, Lyon, France) (13, 14). Patients undergoing human leukocyte antigen (HLA)-matched HSCT were conditioned with busulfan (Bu) and CY. Patients undergoing haploidentical HSCT (haplo-HSCT) were conditioned with Semustine, arabinosylcytosine (Ara-c), Bu, CY, and ATG. The prophylaxis for GVHD included mycophenolate mofetil (MMF), cyclosporin A (CsA), and short-term methotrexate (MTX) (15–17). Supportive care was given according to the institutional standard operating procedures.

### Statistical Analysis

Differences in the white blood cell (WBC) and PB CD34+ cell counts were evaluated by paired t tests. OS and PFS were estimated using the Kaplan–Meier method. Descriptive parameters of the clinical characteristics and outcomes are presented as medians, ranges, and frequencies (%) in the tables. Data were analyzed using SPSS software version 21.0.

## Results

### Donor and Patient Characteristics

The characteristics of donors and patients were listed in Table 1. There were more males than females among the donors. The median donor age was 36 years, which was older than that median patient’s age, but the median donor weight was higher than the median patient weight. HLA-matched HSCT was performed in 43 cases, and haplo-HSCT was performed in 103 cases. Most diseases were hematological malignancies, accounting for 87% of the cases in our study.

**TABLE 1:** Characteristics of donors and patients.

**Donors characteristics**		**Patients characteristics**	
**Parameter**	***n* = 146**	**Parameter**	***n* = 146**
Median age, year (range)	36 (18–59)	Median age, year (range)	26 (2–55)
Males/females, *n*	99/47	Males/females, *n*	91/55
Median weight (kg, range)	63(45–89)	Median weight (kg, range)	55 (5–89)
HLA match, *n*		Disease type, *n*	
Matched	43	AL	104
9/10 matched	2	AA	15
8/10 matched	3	Thalassemia	4
7/10 matched	13	NHL	4
6/10 matched	8	HPS	1
5/10 matched	77	CMML	1
Donor-recipient gender match, *n*		MDS	14
Female–female	17	MS	2
Female–male	29	MM	1
Male–female	38		
Male–male	62		
ABO match, *n*			
Matched	86		
Major mismatched	27		
Minor mismatched	24		
Major-minor mismatched	9		

*AL, acute leukemia; AA, aplastic anemia; MDS, myelodysplastic syndrome; NHL, non-Hodgkin’s lymphoma; HPS, hemophagocytic syndrome; CMML, chronic myelomonocytic leukemia; MS, myeloid sarcoma; MM, multiple myeloma.*

### Mobilization

There was no difference in the WBC count of donors between days 4 and 5 (*P* = 0.53). The median WBC count was 44.79 × 10^9^ L (range, 21.33–100.58 × 10^9^ L) on day 4 and 46.10 × 10^9^ L (range, 19–80.87 × 10^9^ L) on day 5. However, the maximum number of circulating CD34+ cells in donors occurred on day 5 and was significantly higher than that in donors on day 4. The median number of CD34+ cells in the PB of donors was 82.26 μL (range, 9.22–199.7) on day 5 and 51.65 μL (range,10.7–191.10) on day 4 (*P* < 0.001).

Of all donors, only eight donors received an additional daily dose of 10 μg/kg G-CSF because the number of CD34+ cells was ≤20 μL on day 4. The number of CD34+ cells reached 4 × 10^6^ kg in two of the eight donors in a single apheresis procedure.

The median number of monocytes was 27% in the apheresis product. The median number of mononuclear cells and CD34+ cells were 9.4 × 10^8^ and 6.85 × 10^6^ per kg of patient body weight, respectively. In 111 out of 146 (76.0%) donors, an optimal number of CD34+ cells (4 × 10^6^ per kg of patient body weight) was collected in a single apheresis procedure. The minimum collection target, 2 × 10^6^ CD34+ cells/kg, was reached in 91.8% (134/146) of donors in a single apheresis procedure.

### Side Effects of Mobilization

The common side effects of pegfilgrastim treatment were bone pain and headache (Table 2). Most donors had mild pain. No patients had symptoms of abdominal pain and distention. In addition, no cases of splenomegaly were found on examination. Two donors experienced mild thrombocytopenia, and there was a slight increase in the platelet count of these two donors. Increased alkaline phosphatase (ALP) was detected in 91.2% donors, and median ALP was 187.9 U/L. Increased lactate dehydrogenase occurred in all donors, and median lactate dehydrogenase was 506.85 U/L. Increased liver enzymes and lactate dehydrogenase was transient and returned to normal levels by a median of 5 days (range, 3–12 days) after peg-G-CSF administration. The median follow-up for donors was 773 days (range, 552–1269 days). Twenty-two donors showed values exceeding 60 × 10^9^ L; those of 18 donors recovered after 1 week, and those of four donors recovered after 2 weeks. A total of 131 donors were followed up after apheresis. The WBC count of most donors dropped to a normal level within 1 week after apheresis; that of fourteen donors recovered after 2 weeks, and that of six donors recovered after 3 weeks.

**TABLE 2:** Side effects of pegfilgrastim.

**Symptom**	**Intensity**	***N* (%)**
Headache	Total	26 (17.8)
	Mild	26 (17.8)
	Moderate	–
Bone pain	Total	45 (30.8)
	Mild	42 (28.8)
	Moderate	3 (2)
Other complaints	Sleeplessness	9 (6.2)
	Rhinobyon	5 (3.4)
	Sweating	4 (2.7)
	Tiredness	5 (3.4)
	Thrombocytopenia	2 (1.4)
	Thrombocytosis	2 (1.4)

### Engraftment and GVHD

In 140 out of 146 patients (96.9%), neutrophil and platelet engraftment was achieved. Three patients died before engraftment because of severe infection, and only neutrophil engraftment was achieved in one thalassemia patient and two aplastic anemia patients. All patients received elemental blood infusions during transplantation. The median volume of erythrocyte transfusion was 800 ml, and the median volume of platelet transfusion was 1000 ml. The median time for neutrophil and platelet engraftment was 14.5 days (range, 9–33 days) and 15 days (range, 11–84 days), respectively. Patients were monitored for a median of 464 days (range, 31–1077 days). Thirty-seven recipients (26.2%) developed aGVHD, and 53 (36.7%) patients developed cGVHD.

### Non-relapse Mortality, Relapse, and Survival

**FIGURE 2:**
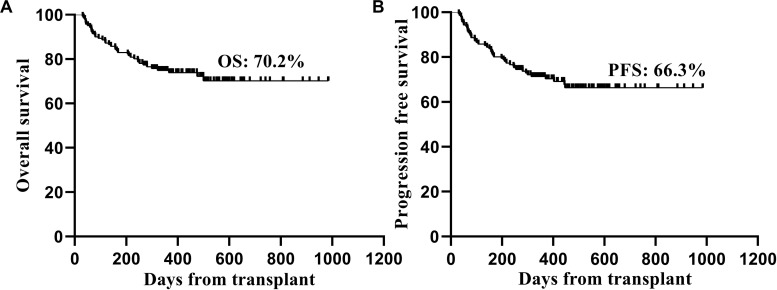
Kaplan-Meier estimated overall survival **(A)** and progression free survival **(B)** of the patients after transplantation.

Thirty-eight patients died after transplantation. The main causes of NRM included GVHD in 17 patients and severe infection in 6 patients (Table 3). Twelve patients died of relapse, and 11 of these patients relapsed within 1 year after transplantation. The 2-year OS and PFS rates after transplantation were 70.2 and 66.3%, respectively (Figures 2A,B).

**TABLE 3:** The summary of death.

**Death**	***N* (%)**
Relapsed	11 (28.9)
GVHD	17 (44.7)
Infection	6 (15.8)
Hemorrhage	2 (5.3)
Cardiac arrhythmia	1 (2.6)
Thrombotic microvascular disease	1 (2.6)

## Discussion

The number of CD34+ cells is critical to the success of transplantation. The optimal number of CD34+ cells can result in faster engraftment and a lower incidence of infection. A total of 4 to 5 × 10^6^ CD34+ cells/kg was recommended as the optimal number of cells by the American Society for Blood and Marrow Transplantation. A retrospective trial in patients with multiple myeloma (MM) showed that patients who received 12 mg of pegfilgrastim were capable of mobilizing a sufficient number of CD34+ cells compared with patients who received G-CSF (18). The administration of single-dose pegfilgrastim in healthy volunteers has also been shown to induce a sufficient increase in CD34+ cells in the PB with kinetics similar to that of conventional G-CSF (19). However, there is still insufficient evidence for pegfilgrastim mobilization. Previous researches showed that donors were treated with single doses of 6–15 mg pegfilgrastim-induced mobilization, and most of them chose 12 mg pegfilgrastim because of the efficiency and steady-state mobilization (7–9). Therefore, we designed this multicenter study to preliminarily evaluate the feasibility and safety of hematopoietic stem cells (HSC) mobilization with 12 mg of pegfilgrastim in healthy donors.

The 146 healthy donors who were enrolled received a single subcutaneous injection of pegfilgrastim (12 mg) at four HSCT centers in China. Haploidentical donors were the main sources in transplantation. Age and sex can affect the mobilization of CD34+ cells. In allogeneic donors, younger male patients are associated with a higher yield of CD34+ cells (20, 21). Therefore, in the case of similar HLA compatibility, we preferred male donors in our study.

The different kinetics of circulating CD34+ cells could have consequences for the scheduling of apheresis procedures. Different mobilization methods cause different kinetics of CD34+ cell circulation (8, 9), and the best collection time should be based on the kinetics. Chanswangphuwana et al. (9) reported that 15 normal allogeneic donors were treated with pegfilgrastim (12 mg) for mobilization, and they found that the maximum concentration of circulating CD34+ cells occurred on day 4, was nearly equal on day 5, and gradually declined on day 6. However, in our study, the peak concentration of CD34+ cells in the PB was detected on day 5, and the number of CD34+ cells was significantly higher on day 5 than on day 4. This finding is consistent with that of Kroschinsky’s study (7). Therefore, day 5 is the best time to collect SCs from healthy donors after pegfilgrastim-induced mobilization.

High doses can result in faster engraftment and can reduce the rates of infection and NRM. However, beyond a certain threshold, there may be no added benefit and a possible increased risk of GVHD (22, 23). Therefore, a CD34+ cell dose between 4 and 5 × 10^6^ CD34+ cells/kg seems optimal based on the available data (4). From most donors (76%) mobilized with pegfilgrastim for mobilization in our study, an optimal number of cells (4 × 10^6^ CD34+ cells/kg) was collected in a single apheresis procedure. Hill et al. (8) compared mobilization using pegfilgrastim and conventional G-CSF. Nineteen donors were mobilized with standard G-CSF for mobilization, and 68.0% of donors yielded >4 × 10^6^ cells/kg patient weight in a single aphaeresis procedure; however, in 12 of 13 donors (92.3%) who received 12 mg pegfilgrastim, >4 × 10^6^ CD34+ cells/kg were collected in a single apheresis procedure. Both of these results indicated that pegfilgrastim-induced mobilization in healthy donors was effective and may even be superior to G-CSF-induced mobilization.

Bone pain, headache, fatigue, nausea, and myalgia are frequent adverse events in HSC mobilization with G-CSF (24). In our study, bone pain and headache were the main side effects of pegfilgrastim. These side effects were transient and usually mild to moderate in severity. Among the 146 donors, only two donors had grade 1 thrombocytopenia; neither donor transfused platelets, and SC collection was not affected. Thrombocytopenia was also reported in another pegfilgrastim-induced mobilization study, and platelet nadirs remained at acceptable levels (21). Nevertheless, a case of severe thrombocytopenia caused by G-CSF in a 14-year-old healthy donor was reported, and HSCs were collected after platelet transfusion (25). This effect was not specific to peg-G-CSF compared with G-CSF. This may have been related to the insufficient expression of proliferation-related genes, such as PF4 and PTFN4 in megakaryocytes (26). It is necessary to closely monitor donor blood parameters during mobilization with G-CSF or pegfilgrastim.

The WBC count usually returned to baseline on day 12 or day 13 after G-CSF-induced mobilization (27, 28). The long half-life of pegfilgrastim may raise the concern of excessive leukocytosis in healthy donors at steady state. However, in our study, the maximum WBC count occurred on days 4–5 and declined during the following days. The WBC count of most donors dropped to a normal level within 1 week after apheresis. We did not observe prolonged postdonation leukopenia. One explanation could be that pegfilgrastim can be eliminated by cellular uptake through the G-CSF receptor and through intracellular degradation, as well as by cleavage by neutrophil elastase, when the number of granulocytes increases (19, 29). There was no cases of splenic rupture or thrombosis in our study or in other pegfilgrastim-induced mobilization studies. Splenomegaly was not detected after PEG mobilization in our observation. However, a donor who received G-CSF was reported to have spleen rupture with WBC count exceeding 50 × 10^9^ L at the time of the event (30). Physicians still need to be aware of this side effect when using pegfilgrastim in normal donors.

In our study, patients transplanted with SC grafts mobilized with pegfilgrastim achieved neutrophil and platelet engraftment after a median of 14.5 and 15 days, respectively, which was similar to the results of other studies of pegfilgrastim- and G-CSF-induced mobilization (9). GVHD is still one of the major causes of morbidity and mortality in allograft recipients, with a high incidence of 30–50% and a 14% mortality rate (31). We observed that the incidence of aGVHD was 26.3%. Other pegfilgrastim studies have reported that GVHD developed in 6.7 to 50% of patients (7, 9). This difference may be associated with the baseline values of the enrolled patients and the limited sample size. cGVHD occurred in 36.7% of patients, which is comparable to values reported by the NIH (30–70%). Morris et al. (32) demonstrated that mobilization with pegfilgrastim results in the enhanced expansion of tolerogenic antigen presenting cells and the augmentation of regulatory T-cell activity, which in turn reduces GVHD. After SC mobilization with pegfilgrastim, graft-versus-leukemia (GVL) and GVHD are effectively separated, and maximal GVL effects are dependent on the presence of invariant natural killer T cells (33, 34). Pegfilgrastim was markedly superior to standard G-CSF for the prevention of GVHD following allogeneic SCT in a murine model (32), and clinical data on HSCT will need to be studied and verified.

## Conclusion

Pegfilgrastim-induced mobilization could be used to collect an optimal number of CD34+ cells in a single procedure, and mild side effects that are similar to those of G-CSF. The collection time of apheresis began on the fifth day, when the highest number of CD34+ cells was observed. The sample size of our study was relatively large. However, this was a retrospective study. A larger randomized study will be necessary to directly compare the effectiveness of pegfilgrastim and G-CSF for mobilization.

## Data Availability Statement

The raw data supporting the conclusions of this article will be made available by the authors, without undue reservation.

## Ethics Statement

The studies involving human participants were reviewed and approved by Xinqiao Ethics Committee. The patients/participants provided their written informed consent to participate in this study.

## Author Contributions

LiG and XZ conceived and designed the study. JL, SW, XZ, and LiG developed the methodology and analyzed and interpreted the data. JL wrote the manuscript. YL, SW, CZ, YZ, SL, PK, LeG, and XZ reviewed and revised the manuscript. All authors contributed to the article and approved the submitted version.

## Conflict of Interest

The authors declare that the research was conducted in the absence of any commercial or financial relationships that could be construed as a potential conflict of interest.
